# Research advances on NLRP3 inflammasomes in organ transplantation

**DOI:** 10.3389/fimmu.2026.1726601

**Published:** 2026-02-09

**Authors:** Kun Wang, Hong Luo, Xiao-jie Ma, Yu Zhang, Yu-xiang Chen, Tao Li, Yi Wang, Hong-tao Jiang

**Affiliations:** 1 Department of Renal Transplantation, The Second Affiliated Hospital of Hainan Medical University, Haikou, China; 2 Department of Rehabilitation Therapy, The Second Affiliated Hospital of Hainan Medical University, Haikou, China

**Keywords:** caspase-1, damage-associated molecules, ischemia-reperfusion injury, NLRP3 inflammasome, organ transplantation

## Abstract

Organ transplantation is a life-saving therapy for end-organ failure; however, long-term outcomes are limited by complications such as ischemia-reperfusion injury (IRI), allograft rejection, and infection. The NLRP3 inflammasome, a key innate immune signaling platform, plays a central role in driving inflammation in these settings. Its activation follows a two-signal paradigm and contributes critically to tissue damage during IRI, bridges innate and adaptive immunity in acute and chronic rejection, and exerts context-dependent roles, either protective or detrimental, during infection. Although targeting NLRP3 through genetic, pharmacological, or cellular approaches shows therapeutic promise in preclinical studies, clinical translation remains challenging. Future efforts should focus on refining these strategies and elucidating its interplay within broader immune networks to improve transplant outcomes.

## 1 Introduction

Organ transplantation, a revolutionary breakthrough in modern medicine, has become a core therapeutic approach for saving lives in patients with end-stage failure of vital organs such as the heart, liver, and kidneys. Since the first successful kidney transplant, advancements in surgical techniques, iterations of immunosuppressive agents, and optimized perioperative management have significantly improved short-term survival rates and quality of life for transplant recipients (1). According to global organ transplant registry data, the 1-year survival rate for kidney transplant recipients now exceeds 95% (2), while the 5-year survival rate for liver transplant recipients remains stable above 70% (3). This medical achievement has brought renewed hope to countless patients facing terminal illness.

However, the three core postoperative complications, ischemic reperfusion injury (IRI), acute/chronic rejection, and secondary microbial infection, remain critical bottlenecks constraining graft long-term survival and recipient prognosis (4–6). Ischemia-reperfusion injury, an unavoidable pathological event during transplantation, can cause early graft dysfunction. Its incidence is particularly pronounced in liver transplantation, with severe cases directly leading to primary graft failure (7, 8). Rejection, representing the immune system’s recognition and attack of the “foreign organ,” occurs in a small proportion of recipients within the first year post-surgery despite potent immunosuppressive therapy. The immunosuppression required for long-term organ preservation compromises immune function, exposing recipients to a higher risk of infection than the general population. With expanding age restrictions and inclusion of more severely ill patients, the indications for organ transplantation continue to broaden (9), further increasing the incidence of post-transplant infectious complications.

In-depth studies reveal that the development of these complications is closely linked to uncontrolled inflammatory responses. As a vital component of innate immunity, inflammation initiates tissue repair mechanisms during the early post-transplant phase (10). However, excessive activation triggers an “inflammatory storm” (11), leading to graft tissue structural damage, vascular endothelial injury, and disruption of immune tolerance (10). Within this intricate inflammatory regulatory network, inflammasomes, multiprotein complexes assembled by intracellular pattern recognition receptors, serve as central regulators (12). By sensing danger signals in the transplant microenvironment, such as ATP released from damaged cells or transplant-associated oxidative stress products, it activates caspase-1, thereby promoting the maturation and release of proinflammatory cytokines IL-1β and IL-18, triggering a cascade of inflammatory responses (13). Among the more than ten inflammasomes identified, NLRP3 has emerged as a research hotspot in organ transplantation due to its diverse activation mechanisms (responding to metabolic disturbances, oxidative stress, pathogen invasion, and other stimuli) and high expression in multiple transplant-related disease models. Numerous animal studies confirm that inhibiting NLRP3 inflammasome activity significantly reduces IRI severity after heart transplantation (14), diminishes inflammatory responses and apoptosis in kidney transplantation (15), and decreases infection complications following lung transplantation. These findings provide novel insights into the molecular mechanisms of transplant complications and lay a theoretical foundation for developing novel inflammasome-targeted therapeutic strategies.

Therefore, systematically elucidating the mechanisms of NLRP3 inflammasome in organ transplantation and clarifying its regulatory networks in IRI, rejection, and infection holds significant theoretical value and clinical translational implications for overcoming current therapeutic bottlenecks and achieving long-term graft survival.

## 2 Structure and activation mechanism of the NLRP3 inflammasome

### 2.1 Structural features and molecular assembly mechanism of the NLRP3 inflammasome

**Figure 1:**
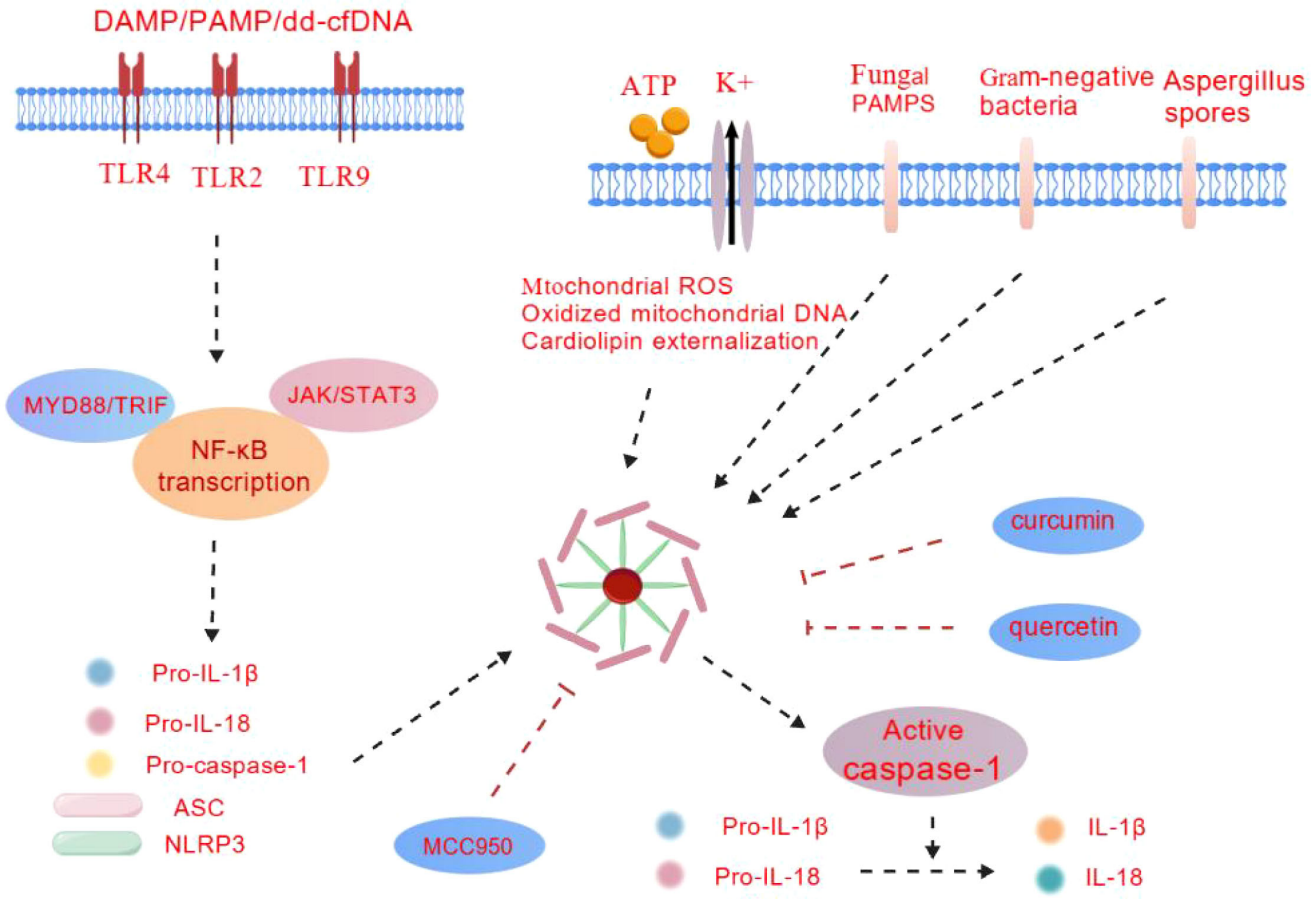
NLRP3 inflammasome signaling in transplantation. This figure illustrates the molecular mechanism of NLRP3 inflammasome activation, with a focus on the molecular mechanisms unique to solid organ transplantation. Extracellular ATP activates receptors, promotes potassium efflux, and releases oxidized mitochondrial DNA and dd cfDNA, leading to increased mitochondrial ROS and cardiolipin externalization. This in turn promotes NLRP3 inflammasome oligomerization and activation, thereby releasing IL-1 β, IL-18, and self catalyzing cleavage of caspase-1. Bacterial and fungal infections during transplantation: PAMP and/or DAMP (such as dd cfDNA) activate TLRs, promoting the activation of regulatory NLRP3. NLRP3 transcription steps: The transcription steps are regulated by TLRs - MyD88, JAK/STAT, which promote NF - κ B activation and induce transcription of pre-IL-1- β, NLRP3, and caspase-1. MCC950、 Quercetin and curcumin can exert protective effects in organ transplantation and transplant related injuries by inhibiting the aggregation or assembly of NLRP3 inflammasomes.

As one of the most extensively studied members of the NOD-like receptor (NLR) family, the NLRP3 inflammasome serves as a key molecular platform regulating innate immune responses and inflammatory reactions (**Figure 1**). Its core structure comprises the NLRP3 protein, apoptosis-related spot-like protein (ASC), and caspase-1 (Caspase-1). These three components form a functional complex through precise domain interactions, playing an irreplaceable role in recognizing danger signals and initiating inflammatory cascades.

#### 2.1.1 Domain composition and functional characteristics of NLRP3 protein

As the core recognition unit of the inflammasome, the NLRP3 protein exhibits a typical three-domain molecular structure, with each domain performing specific functions in signal sensing and complex assembly.

The N-terminal pyrophosphorylated domain (PYD) belongs to the death domain superfamily, comprising a conserved folding structure of six α-helices. This domain forms a stable heterodimer through homodimeric interactions with the PYD domain of ASC protein via a conserved hydrophobic interface, constituting the initial molecular event in inflammasome assembly (16, 17). Studies indicate that mutations in key amino acid residues within the α2 and α3 helical regions of the PYD domain significantly inhibit NLRP3-ASC binding efficiency, thereby blocking downstream signaling (18).

The intermediate nucleotide-binding and oligomerization domain (NACHT) is a hallmark domain of the NLR family, containing conserved Walker A and Walker B motifs and possessing ATPase activity (19). In the resting state, the NACHT domain maintains an autoinhibitory conformation through intramolecular interactions with the LRR domain. Upon activation by signals, its ATPase activity is triggered. The binding and hydrolysis of ATP drive NLRP3 to undergo conformational rearrangement, releasing the autoinhibition and mediating protein oligomerization to form a functional polymeric scaffold (20). Recent cryo-EM studies confirm that activated NLRP3 forms oligomers with hexagonal symmetry, providing a spatial platform for subsequent recruitment of adaptor proteins.

The C-terminal leucine-rich repeat (LRR) domain comprises multiple leucine repeat units, forming a curved horseshoe structure. As the core region for pattern recognition, this domain identifies various pathogen-associated molecular patterns (PAMPs) and damage-associated molecular patterns (DAMPs) through conformational changes (21). Notably, the LRR domain not only participates in danger signal recognition but also regulates the activation threshold of NLRP3 through dynamic interactions with the NACHT domain. This self-regulatory mechanism constitutes a crucial molecular basis for maintaining immune homeostasis.

#### 2.1.2 The bridging function and structural basis of ASC

Apoptosis-related spot-like protein (ASC), a key signaling adaptor molecule, mediates functional coupling between NLRP3 and Caspase-1 through a molecular bridging action facilitated by its dual domains (22).

The ASC molecule comprises an N-terminal PYD domain and a C-terminal caspase recruitment domain (CARD), connected by a flexible linker. This structural feature enables simultaneous recognition and binding of upstream sensors and downstream effector molecules (23). Its PYD domain forms a stable complex with the PYD domain of NLRP3 through a specific hydrogen bond network, while the C-terminal CARD domain undergoes homodimeric interaction with the CARD domain of Caspase-1, establishing a continuous signaling pathway (24).

During inflammasome assembly, ASC not only serves as a linker but also forms fibrillar aggregates via the aggregation properties of its own PYD domain. This “speckle-like” supramolecular structure significantly increases local protein concentration, promoting the oligomerization and activation of Caspase-1. Recent studies reveal that ASC fibers exhibit a core-shell structure: the core consists of a fiber scaffold formed by PYD-PYD interactions, while the shell comprises an ordered arrangement of CARD domains. This structural arrangement efficiently mediates effector molecule activation (25).

#### 2.1.3 Activation mechanism and substrate specificity of caspase-1

As an effector molecule of inflammasomes, Caspase-1 belongs to the cysteine protease family. Its activation process involves precise conformational changes and proteolytic events (26).

In its inactive state, Caspase-1 exists as a pro-enzyme (pro-Caspase-1), comprising an N-terminal CARD domain, a large subunit (p20), and a small subunit (p10) (27). Upon forming a complex with ASC and NLRP3, pro-Caspase-1 undergoes oligomerization through CARD domain interactions, inducing intramolecular proteolysis and releasing the catalytically active p20/p10 heterodimer. Activated Caspase-1 specifically cleaves substrate proteins at aspartic acid residues through nucleophilic attack on cysteine residues (28). Its primary physiological substrates include pro-IL-1β and pro-IL-18; cleavage by Caspase-1 removes the N-terminal precursor peptide, yielding mature IL-1β and IL-18 (29). These active cytokines are released extracellularly via non-classical secretory pathways, initiating downstream inflammatory cascades that recruit and activate immune cells, thereby enhancing local immune responses (30). Notably, Caspase-1 activation is also closely associated with pyroptosis. By cleaving substrate molecules such as gasdermin D, it mediates inflammatory cell death, a process crucial for clearing intracellular pathogens (31).

#### 2.1.4 Assembly kinetics and structural regulation of the inflammasome

The assembly of the NLRP3 inflammasome is a highly ordered dynamic process involving multiple structural transitions and molecular recognition events to form a complete signaling pathway.

During the initiation phase, LRR domain recognition of danger signals triggers NLRP3 conformational rearrangement, activating the ATPase activity of the NACHT domain. This drives protein oligomerization to form a primary complex. Subsequently, oligomerized NLRP3 recruits ASC molecules via PYD-PYD interactions, forming an intermediate complex. ASC molecules self-assemble into fibrillar structures while recruiting pro-Caspase-1 via their CARD domains, ultimately forming the mature inflammasome complex (**Figure 1**). Within this complex, pro-Caspase-1 undergoes self-cleavage activation, initiating the maturation and release of downstream inflammatory cytokines (32).

This assembly process undergoes multi-level regulation influenced by factors including post-translational modifications (phosphorylation, ubiquitination, etc.), ion concentration changes, and cellular metabolic states. Elucidating the structural characteristics and assembly mechanisms of the NLRP3 inflammasome not only advances our understanding of the molecular basis of innate immunity but also provides critical structural biology insights for targeted therapies in inflammation-related diseases.

### 2.2 Activation mechanism and regulatory network of the NLRP3 inflammasome

The activation of the NLRP3 inflammasome is a finely regulated multistep process, characterized by a “dual-signal activation model” (33). This model employs hierarchical signal regulation to ensure rapid inflammatory responses to danger signals while maintaining strict activation thresholds to prevent tissue damage from excessive inflammation. In recent years, the molecular details of this activation mechanism have been progressively revealed through techniques such as single-molecule imaging and cryo-electron microscopy.

#### 2.2.1 Signal 1: molecular conduction pathway of initiating signals

Signal 1 serves as the preparatory step for inflammasome activation, primarily initiating the transcriptional expression of relevant molecules through pattern recognition receptor-mediated signaling pathways, thereby laying the material foundation for subsequent activation.

The Toll-like receptor (TLR) family serves as the primary receptor for transmitting the initiation signal. Specifically, TLR4 recognizes lipopolysaccharide (LPS), TLR3 detects viral double-stranded RNA, TLR2 identifies bacterial peptidoglycan, and other pathogen-associated molecular patterns (PAMPs), while TLR9 recognizes damage-associated molecular patterns (DAMPs) such as endogenous DNA (34). Upon ligand binding, these receptors initiate downstream signaling cascades by recruiting intracellular adaptor proteins MyD88 or TRIF: MyD88 recruits IRAK4 and IRAK1 to form a signaling complex, activating TAK1 kinase, which subsequently phosphorylates the IKK complex (IKKα/β/γ). This leads to the phosphorylation and degradation of the NF-κB inhibitor IκBα, releasing the NF-κB dimer (p65/p50) for translocation to the nucleus, where it initiates target gene transcription (35).

This transcriptional regulation primarily promotes the expression of three types of molecules: NLRP3 protein itself, pro-inflammatory cytokine precursors pro-IL-1β and pro-IL-18, and certain inflammasome regulatory molecules. Notably, gene expression profiles induced by different activation signals exhibit distinct patterns: bacterial PAMPs predominantly upregulate NLRP3 and pro-IL-1β expression, whereas viral infection more significantly promotes pro-IL-18 transcription. This selective regulation may correlate with distinct pathogen immune evasion strategies (36, 37). Recent studies reveal that Signal 1 also modulates chromatin accessibility in the NLRP3 promoter region via epigenetic modifications, enabling fine-tuned regulation of its expression levels (38).

#### 2.2.2 Signal 2: molecular trigger mechanism of activation signals

Signal 2 serves as the “trigger signal” for NLRP3 inflammasome assembly, initiating the formation of functional inflammasome complexes by inducing NLRP3 conformational rearrangement and oligomerization (39). Multiple intracellular environmental changes have been confirmed as potential Signal 2s, converging on NLRP3 activation pathways through distinct molecular mechanisms.

Ion homeostasis disruption stands as one of the most well-established activation signals. Among these, potassium efflux is considered the most conserved activation mechanism: when cells are stimulated by ATP (via P2X7 receptors), nigericin, or similar agents, the opening of ion channels in the cell membrane leads to changes in intracellular potassium concentration. This directly disrupts the self-inhibitory interaction between NLRP3 and its LRR domains, promoting conformational rearrangement (40). Recent cryo-EM structures reveal that hypokalemic conditions induce exposure of the ATP-binding site within the NACHT domain of NLRP3, establishing the structural foundation for subsequent oligomerization (41). Additionally, calcium influx participates in NLRP3 activation regulation by activating calmodulin-dependent kinase II (CaMKII), though its precise molecular targets require further elucidation (42).

Reactive oxygen species (ROS) accumulation represents another critical activation signal, primarily originating from mitochondrial respiratory chain dysfunction or NADPH oxidase activation. Mitochondrial ROS can alter NLRP3’s conformation by oxidizing key cysteine residues while promoting mitochondrial DNA (mtDNA) release into the cytoplasm. Released mtDNA directly enhances NLRP3 activation by binding to its LRR domain (43). Studies confirm that the antioxidant N-acetylcysteine (NAC) significantly inhibits urate crystal-induced NLRP3 activation, providing experimental evidence for the critical role of ROS (44).

Lysosomal damage-mediated activation signals are primarily associated with crystalloid substances (e.g., uric acid crystals, asbestos fibers). Upon entering cells via endocytosis, these substances physically disrupt lysosomal membrane integrity, leading to the release of lysosomal proteases (e.g., cathepsin B) into the cytoplasm (45). Cathepsin B releases NLRP3 from its autoinhibited state by cleaving specific sites on NLRP3 or degrading its inhibitory proteins (46).

Mitochondrial dysfunction contributes to activation through multiple mechanisms: beyond releasing ROS and mtDNA, cardiolipin released from damaged mitochondria directly binds to NLRP3, while inhibition of mitophagy leads to accumulation of damaged mitochondria, further amplifying the activation signal (47). Recent studies reveal that the formation of the mitochondria-endoplasmic reticulum contact site (MAM) can recruit NLRP3 and associated signaling molecules, creating localized activation microdomains (48).

#### 2.2.3 Synergistic regulation and spatiotemporal coupling of dual signals

Activation of the NLRP3 inflammasome is not a simple summation of two signals but involves precise spatiotemporal coordination. NLRP3 protein synthesis induced by Signal 1 must reach a threshold concentration to respond to Signal 2. This “dose-dependent” characteristic prevents accidental activation of NLRP3 at low expression levels.

Temporally, Signal 1 typically precedes Signal 2, providing sufficient protein reserves for inflammasome assembly. The duration of Signal 2 determines inflammatory response intensity, brief stimulation triggers localized activation, while sustained stimulation leads to systemic cytokine release.

Spatially, NLRP3 activation occurs within specific intracellular microdomains: at endoplasmic reticulum-mitochondrial contact sites, NLRP3 interacts with the adaptor protein ASC via their PYD domains to form an initial complex, followed by the recruitment and activation of Caspase-1 (49–51). This spatial confinement ensures controlled transmission of inflammatory signals, preventing disruption to core cellular functional zones.

#### 2.2.4 Post-activation regulatory network

Following NLRP3 inflammasome activation, multi-level negative feedback regulation persists to prevent excessive inflammation-induced tissue damage. At the transcriptional level, IL-1β downregulates NLRP3 expression by activating IL-1 receptors. At the post-translational modification level, ubiquitination (e.g., K48-linked ubiquitination mediated by TRIM31) and phosphorylation (e.g., Ser295 phosphorylation mediated by AMPK) promote NLRP3 degradation or inhibit its oligomerization (52, 53). Furthermore, autophagy constitutes a crucial negative feedback loop by selectively degrading activated inflammasome complexes (54).

These intricate regulatory mechanisms collectively maintain the activation equilibrium of the NLRP3 inflammasome, whose dysregulation is closely associated with various inflammatory diseases (e.g., gout, Alzheimer’s disease, atherosclerosis). Elucidating its molecular activation mechanisms provides a critical theoretical foundation for developing specific anti-inflammatory drugs targeting NLRP3.

## 3 Role of NLRP3 inflammasome in organ transplantation-related complications

### 3.1 NLRP3 inflammasome and ischemia-reperfusion injury

The NLRP3 inflammasome plays a pivotal role in IRI-mediated inflammation (55). Ischemia-reperfusion injury (IRI) is a common and severe early complication following organ transplantation, adversely affecting graft functional recovery and long-term survival. During IRI, the ischemic phase induces tissue hypoxia, impaired energy metabolism, disrupted intracellular homeostasis, impaired mitochondrial function, and increased reactive oxygen species (ROS) production. Reperfusion then floods tissues with oxygen molecules, further exacerbating oxidative stress while activating the innate immune system and triggering inflammatory responses (56–59). When donor organs undergo ischemia-reperfusion, factors such as mitochondrial damage and lysosomal leakage stimulate cells to produce abundant DAMPs, including heat shock proteins, mtDNA, and ATP, which activate the NLRP3 inflammasome (60, 61). The activated NLRP3 inflammasome promotes the cleavage of pro-Caspase-1 into active Caspase-1, which cleaves pro-IL-1β and pro-IL-18, leading to their maturation and release into the extracellular space. This triggers downstream inflammatory cascades, resulting in tissue injury and organ dysfunction (62–64).

In kidney transplantation, IRI causes damage to renal tubular epithelial cells, microvascular dysfunction, and inflammatory cell infiltration in the renal interstitium. Studies indicate that inhibiting NLRP3 inflammasome activity can mitigate renal IRI injury and improve renal function (65, 66). For instance, in a mouse kidney transplantation model treated with the NLRP3-specific inhibitor MCC950, significant reductions were observed in renal tissue expression of NLRP3, ASC, and Caspase-1. This treatment also decreased IL-1β and IL-18 release, mitigated tubular injury and interstitial inflammation, and improved graft survival (67). In liver transplantation, IRI similarly activates the NLRP3 inflammasome, triggering inflammatory responses that lead to hepatocyte apoptosis and impaired liver function. Inhibiting the NLRP3 inflammasome mitigates hepatic IRI injury and promotes liver function recovery (68). Related studies indicate that knockout of the NLRP3 gene in mice using gene knockout technology significantly reduces inflammatory injury in the liver and markedly improves liver function indicators in a liver transplantation IRI model.

In heart transplantation, IRI can lead to myocardial cell necrosis, myocardial interstitial edema, and arrhythmia. NLRP3 inflammasome is rapidly activated in myocardial IRI, and the released IL-1 β can exacerbate myocarditis infiltration. Inhibiting NLRP3 activity can significantly reduce myocarditis and improve the contractile function of the transplanted heart (69). In lung transplantation, IRI leads to a significant release of intracellular ATP and its conversion into extracellular ATP (eATP), which activates the purinergic receptor P2X7 to initiate NLRP3 inflammasome assembly; After activation, the inflammasome mediates the mature release of IL-1 β and IL-18, inducing neutrophil infiltration on one hand, and triggering apoptosis of alveolar epithelial cells and downregulating the expression of tight junction proteins such as Claudin-5 and Occludin on the other hand, ultimately leading to alveolar barrier disruption and pulmonary edema (70).

### 3.2 NLRP3 inflammasome and rejection reactions

Rejection, representing the immune system’s “recognition-attack” response to the graft post-transplantation, is categorized into three major types based on timing, immune mechanisms, and pathological features: hyperacute rejection, acute rejection, and chronic rejection. As a key cross-linking molecule between innate and adaptive immunity, the NLRP3 inflammasome plays a crucial role in the initiation, amplification, and maintenance of various rejection responses. Its activation mechanisms and pathological effects exhibit distinct stage-specificity (71).

Hyperacute rejection, a severe immune response occurring within minutes to hours after transplantation, is primarily triggered by the binding of recipient pre-existing antibodies (such as anti-HLA antibodies and ABO blood group antibodies) to antigens on the surface of graft vascular endothelial cells. This process is accompanied by rapid activation of the complement system and thrombosis. Recent studies reveal that the NLRP3 inflammasome exacerbates tissue injury through the “complement-NLRP3 axis”: C5a fragments generated by complement activation directly bind to C5aR on neutrophil surfaces, triggering intracellular potassium efflux and reactive oxygen species bursts (71, 72), thereby activating the NLRP3 inflammasome. The latter, by releasing IL-1β and IL-18, further recruits neutrophil infiltration and enhances the procoagulant activity of vascular endothelial cells, forming a vicious cycle of “complement activation - inflammasome activation - thrombosis” (73–76). Studies indicate that inhibiting the NLRP3 inflammasome delays the onset of hyperacute rejection and reduces intravascular thrombus area in grafts, suggesting it may serve as a potential target for blocking hyperacute rejection in xenotransplantation.

Acute rejection typically occurs within days to months post-transplantation, characterized by T cell-mediated immune attacks accompanied by extensive inflammatory cell infiltration and graft parenchymal injury. NLRP3 inflammasome exerts a “dual priming effect” during this phase: On one hand, residual damage-associated molecular patterns (DAMPs) in the graft (e.g., heat shock proteins, ATP) directly activate NLRP3 inflammasomes within resident macrophages and dendritic cells (DCs). The released IL-1β promotes T cell activation by upregulating DC surface co-stimulatory molecules (CD80/CD86) (77). On the other hand, IFN-γ secreted by activated Th1 cells enhances NLRP3 inflammasome assembly efficiency in macrophages, forming a cascade reaction of “innate immune initiation - adaptive immune amplification” (77). The NLRP3 inflammasome connects innate and adaptive immunity through the maturation of IL-1β and IL-18; IL-1β promotes Th17 cell differentiation (associated with IL-17 production) and recruits CD4^+^T and CD8^+^T cells to the graft site. In heart transplantation, inflammasome activation has been described as a key driver of “sterile inflammation,” participating in the initiation of the adaptive immune response.

Chronic rejection, a major barrier to long-term graft survival, is characterized by chronic graft fibrosis and occlusive vascular lesions, with disease progression spanning years to decades. The NLRP3 inflammasome participates in this pathological process through a “low-level persistent activation” model: on one hand, gut microbiota dysbiosis induced by long-term immunosuppressive therapy can persistently activate the NLRP3 inflammasome via metabolic byproducts (e.g., reduced short-chain fatty acids, translocated lipopolysaccharides), promoting macrophage polarization toward a pro-fibrotic phenotype (78, 79); On the other hand, continuous stimulation of vascular endothelial cells by oxidized LDL and mechanical stress activates NLRP3 inflammasomes, which promote smooth muscle cell proliferation and migration via the IL-1β/IL-18 signaling axis, accelerating the progression of chronic allograft nephropathy (CAN) in kidney transplantation and cardiovascular allograft disease (CAV) in heart transplantation (80). Studies indicate that serum levels of NLRP3 inflammasome-associated markers (e.g., caspase-1 active fragment, IL-1β) in models of chronic rejection post-kidney transplantation are elevated compared to stable-phase recipients and positively correlate with glomerular sclerosis severity, suggesting its potential as an early warning indicator and intervention target for chronic rejection (81, 82). In the process of fibrosis, NLRP3 can exert its effects through three specific pathways. Firstly, through the IL-1 β/MyD88/TGF - β/Smad axis, it activates downstream complexes and initiates TGF - β 1 transcription and secretion by binding to IL-1 β receptors, thereby activating the Smad pathway to induce ECM related gene expression. This triggers EMT and IF/TA in renal tubular epithelial cells during kidney transplantation, and activates hepatic stellate cells during liver transplantation, leading to liver parenchymal fibrosis (83), Secondly, through the IL-18/JAK/STAT3/CTGF axis, IL-18 activates the JAK/STAT3 pathway to upregulate CTGF, amplify ECM synthesis, and inhibit its degradation (84); In terms of vascular occlusive lesions, NLRP3 mainly induces high expression of Jagged1 in vascular endothelium and activates the Notch pathway of VSMC through the IL-1 β/IL-18/Notch/Jagged1 axis, promoting phenotype transformation and proliferation migration, leading to thickening of vascular endothelium (85); Through the IL-1 β/PI3K/Akt/mTOR axis, PI3K/Akt is activated and mTORC1 and FoxO3a are regulated, promoting VSMC proliferation and inhibiting cell autophagy, especially leading to stenosis of renal transplant artery anastomosis. EndMT can also be induced through multiple pathways, disrupting the endothelial barrier and anticoagulant function, accelerating thrombus formation and intimal hyperplasia (86).

In summary, the NLRP3 inflammasome participates in the pathological processes of three types of rejection through distinct molecular mechanisms. Its specific regulation holds promise for providing novel strategies for the precise prevention and treatment of transplant rejection and prolonging graft survival.

### 3.3 NLRP3 inflammasome and microbial infection

As a crucial sensor in innate immunity, the NLRP3 inflammasome plays a dual role in the anti-infective immunity of organ transplant recipients: on one hand, it recognizes invading pathogens such as viruses and bacteria, initiating inflammatory responses to eliminate pathogens; on the other hand, under immunosuppressed conditions, its excessive activation or dysregulation may exacerbate infection-related tissue damage and even induce systemic inflammatory response syndrome (87, 88). This complex mechanism renders it a key target in post-transplant infection prevention research.

#### 3.3.1 Role in viral infections

Organ transplant recipients exhibit significantly heightened susceptibility to viruses due to long-term immunosuppressive therapy, with cytomegalovirus (CMV), herpes simplex virus (HSV), and influenza virus being common pathogens. The NLRP3 inflammasome activates immune responses by recognizing viral nucleic acids or replication products. For example, during CMV infection, the release of intracellular mitochondrial DNA triggers NLRP3 inflammasome assembly, activating caspase-1 and releasing IL-1β and IL-18 (89), which in turn recruit natural killer cells and T cells to eliminate infected cells. Studies indicate that in CMV-positive kidney transplant recipients, peripheral blood monocyte NLRP3 expression correlates positively with viral load (90), suggesting its role in monitoring antiviral immunity.

However, viruses have evolved escape mechanisms to interfere with NLRP3 function. For instance, the influenza virus non-structural protein NS1 directly binds NLRP3 to inhibit its oligomerization, resulting in weakened inflammatory responses and delayed viral clearance. This “host-virus” game creates a regulatory dilemma for NLRP3 inflammasomes: overactivation may trigger a “cytokine storm” (e.g., acute respiratory distress syndrome following influenza infection), while underactivation fails to effectively control viral replication.

#### 3.3.2 Role in bacterial infections

Bacterial infections are the most common post-transplant infections. Both Gram-negative bacteria (e.g., Escherichia coli, Klebsiella pneumoniae) and Gram-positive bacteria (e.g., Staphylococcus aureus, Enterococcus) activate the NLRP3 inflammasome through distinct mechanisms. For Gram-negative bacteria, their cell wall component lipopolysaccharide (LPS) first induces pro-inflammatory factor precursor (pro-IL-1β) expression via the TLR4 signaling pathway. Subsequently, bacterial exotoxins (e.g., Shiga toxin) or ion disruption (e.g., potassium ion efflux) trigger NLRP3 inflammasome activation, forming a “two-step activation model” (91, 92). In contrast, peptidoglycan fragments and toxins released by Gram-positive bacteria directly damage cell membranes, activating the NLRP3 pathway through reactive oxygen species accumulation or lysosomal rupture.

In transplant recipients, this activation mechanism has dual effects: on one hand, NLRP3-mediated inflammation enhances neutrophil phagocytosis, controlling early bacterial spread (e.g., in Klebsiella pneumoniae urinary tract infections); on the other hand, prolonged immunosuppression may lead to NLRP3 overactivation (93). For instance, during Staphylococcus aureus infections, the phenol-soluble regulatory protein it produces can continuously stimulate NLRP3 to release IL-1β, exacerbating tissue and cellular damage and even inducing sepsis (94, 95). Clinical studies reveal elevated serum IL-1β levels in patients with bacterial infections compared to non-septic individuals, with NLRP3 gene polymorphisms significantly correlated with sepsis susceptibility. Research demonstrates that Acinetobacter baumannii powerfully activates the NLRP3 inflammasome’s nucleotide-binding and oligodomain-containing receptor family pyrin domain through dectin-1/Syk-dependent signaling and the cytoplasmic scaffold protein p62/SQSTM1 (p62) in human macrophages, thereby initiating inflammatory responses (96).

#### 3.3.3 Role in fungal infections

Fungal infections carry high mortality rates in organ transplant recipients, where NLRP3 inflammasomes primarily function by recognizing fungal cell wall components such as β-glucan and mannose. Candida hyphae activate the NLRP3-ASC complex via the Syk kinase pathway, while Aspergillus fumigatus conidia activate NLRP3 through ROS-dependent mechanisms within phagocytes, inducing IL-1β release to recruit inflammatory cells (97).

However, NLRP3 inflammasome activation may be constrained under immunosuppressed conditions: for instance, prolonged glucocorticoid use affects NF-κB expression, thereby suppressing NLRP3 expression and diminishing fungal clearance capacity. This contributes to the progression of post-transplant Aspergillus infection to invasive pulmonary aspergillosis (98). Furthermore, NLRP3 overactivation triggered by fungal infection may exacerbate graft injury. For instance, in kidney transplant recipients with concurrent candidemia, NLRP3-mediated inflammation can induce acute tubular necrosis, causing abrupt decline in graft function (99). In Candida infection, morphological transformation of hyphae is a necessary condition for activating NLRP3. Candidalysin secreted by hyphae can activate NLRP3 by inducing potassium ion efflux and ROS production. At the same time, Dectin-1 recognizes β - glucan to initiate NLRP3 pre activation. Activated NLRP3 can recruit pro-inflammatory factors to activate immune cells and enhance antifungal defense (100); In Aspergillus infection, the galactomannan and hypoxanthine of Aspergillus fumigatus can activate NLRP3 through multiple pathways. Its ROS Syk axis, potassium ion efflux, lysosome disruption, and other pathways promote inflammasome assembly (101), At the same time, NLRP3 can also form a “safety redundancy system” with AIM2, jointly forming a collaborative protection network against Aspergillus (102).

In summary, NLRP3 inflammasomes participate in anti-infective immunity by recognizing specific components of different microorganisms. However, their regulation in transplant recipients requires precise balance, maintaining sufficient immune responses to clear pathogens while avoiding tissue damage caused by excessive activation. Deepening our understanding of its interaction mechanisms with microorganisms may provide a theoretical foundation for developing anti-infective therapeutic strategies targeting NLRP3.

## 4 Regulatory strategies for the NLRP3 inflammasome and its application prospects in organ transplantation

### 4.1 Genetic regulation

Genetic regulation represents a key intervention strategy for the NLRP3 inflammasome. Gene editing technologies, such as the CRISPR/Cas9 system, can block NLRP3 inflammasome activation by knocking out or silencing upstream NLRP3 genes. In relevant experiments, CRISPR/Cas9-mediated knockout of the upstream gene TXNIP in mice resulted in significantly reduced inflammatory responses and improved renal ischemia-reperfusion injury in an IRI model. However, gene editing technologies face numerous challenges in clinical application, including off-target effects, immunogenicity, and ethical concerns, limiting their widespread use. Additionally, RNA interference (RNAi) technology can be employed to silence NLRP3 gene expression. By designing small interfering RNA (siRNA) or short hairpin RNA (shRNA) targeting NLRP3 mRNA, NLRP3 expression levels can be specifically reduced, thereby inhibiting NLRP3 inflammasome activation. In both cellular experiments and animal models, RNAi technology has been demonstrated to effectively suppress NLRP3 inflammasome-related inflammatory responses (103). However, RNAi technology also presents challenges such as low transfection efficiency, poor *in vivo* stability, and potential immune stimulation, necessitating further optimization and refinement.

### 4.2 Pharmacological intervention

Drug intervention is currently the most extensively studied strategy for regulating the NLRP3 inflammasome. Multiple small-molecule compounds have been identified that can inhibit NLRP3 inflammasome activation. MCC950 is a specific NLRP3 inhibitor that binds to the NACHT domain of NLRP3, blocking its oligomerization and activation, thereby suppressing the production of downstream inflammatory cytokines. In organ transplantation-related animal models, MCC950 has demonstrated promising therapeutic effects. For instance, in a renal transplantation ischemia-reperfusion injury (IRI) model, MCC950 treatment significantly reduced renal tissue damage and improved renal function (104). In diabetic nephropathy models, MCC950 also reduced inflammatory responses (105). At present, MCC950 has entered phase I/II clinical trials for some inflammatory diseases, and clinical trials for transplant related diseases are still in the preliminary exploration stage. The core challenges it faces include: how to suppress NLRP3 inflammation while avoiding weakening the anti infective immunity of transplant recipients; How to combine with existing immunosuppressants to avoid drug interactions and reduce low toxicity and side effects; And how to achieve targeted delivery of transplanted organs and increase local drug concentration.Beyond MCC950, other compounds such as glibenclamide, quercetin, and curcumin have also been reported to inhibit NLRP3 inflammasome activity, but they typically lack specificity, and their effects may stem from a wide range of anti-inflammatory and antioxidant properties. Glibenclamide inhibits NLRP3 activation by blocking K^+^ efflux pathways (106); quercetin and curcumin possess antioxidant and anti-inflammatory properties that suppress NLRP3 inflammasome activation through multiple mechanisms (107, 108). These drugs are mostly in the preclinical research stage, and their effectiveness and safety in transplant patients still need to be further validated through large-scale clinical trials. Some drugs have low bioavailability and non-specific targets, and their clinical application value needs to be improved through drug molecular modification.

### 4.3 Cell therapy

As an emerging therapeutic approach, cell therapy also shows potential applications in regulating the NLRP3 inflammasome. Bone marrow-derived mesenchymal stem cells (BMSCs) are among the most extensively studied stem cell types for cell therapy. BMSCs possess multiple functions, including immunomodulation, anti-inflammation, and tissue repair. Studies indicate that BMSCs exert therapeutic effects on organ transplant-related complications by inhibiting NLRP3 inflammasome activation (109). In the field of organ transplantation, BMSC therapy may also function through similar mechanisms. For instance, in transplant ischemia-reperfusion injury (IRI) models, transplanted BMSCs reduce tissue expression of NLRP3, ASC, and Caspase-1, thereby mitigating inflammatory damage and improving organ function (110, 111). Additionally, other cell types such as regulatory T cells (Tregs) mediate immunosuppression through adenosine production via CD39/CD73 expression. Adenosine has been demonstrated to inhibit NLRP3 inflammasome activation by blocking ATP-P2X7 signaling (112). Tregs suppress effector T cell activation and proliferation, reducing inflammatory cytokine production and thereby mitigating inflammatory responses (113). In transplant rejection, adoptive transfer of Tregs has been shown to prolong graft survival, with a potential mechanism involving inhibition of NLRP3 inflammasome-related inflammatory responses (114).

## 5 Conclusions and outlook

The NLRP3 inflammasome plays a key role in complications such as ischemia-reperfusion injury (IRI), rejection, and microbial infections after organ transplantation, and its activation mediated inflammatory response is the core mechanism causing graft injury and functional impairment. In IRI, NLRP3 exacerbates early tissue damage in multiple organ transplantation; In rejection reactions, it can promote the fibrosis and vascular occlusion process of chronic rejection through multiple specific signaling pathways; In infections, the dual effects of immune defense and pathological damage against Candida and Aspergillus are particularly prominent in transplant recipients with immune suppression.

NLRP3 and its downstream cytokines (such as IL-1 β and caspase-1 active fragments) have great potential as biomarkers for predicting or monitoring transplant complications. For example, the levels of relevant biomarkers in the serum of chronic kidney transplant rejection patients are positively correlated with the degree of glomerulosclerosis, and the expression of NLRP3 in peripheral blood mononuclear cells of CMV positive kidney transplant recipients is positively correlated with viral load. In the future, standardized detection systems can be established to achieve early warning of transplant complications.

At present, the regulatory strategies for NLRP3 inflammasome, including gene regulation, drug intervention, and cell therapy, have achieved promising results in animal research. The clinical translation of inhibitors such as MCC950 has entered the preliminary exploration stage, but clinical translation faces significant challenges: gene editing technology has safety and ethical controversies; Drugs need to balance efficacy and toxic side effects, while addressing compatibility issues with existing immunosuppressants; Cell therapy requires the establishment of a standardized preparation and quality control system.To promote the clinical translation of NLRP3 targeted therapy into organ transplantation in the future, priority should be given to optimizing existing regulatory strategies: developing precise gene delivery systems to reduce off target effects; Modify drug molecules to enhance specificity and reduce toxicity, while conducting multicenter, randomized controlled clinical trials to validate their safety and efficacy in transplant patients; Establish a large-scale preparation system for cell therapy. In addition, it is necessary to thoroughly analyze the cross regulatory mechanisms of NLRP3 with immune pathways such as TLR4 and NF - κ B, and construct a multi-target joint regulatory scheme. Meanwhile, emerging technologies such as single-cell RNA sequencing and spatial transcriptomics can help elucidate the cell type specific function of NLRP3 in the transplantation microenvironment, such as distinguishing the differential role of NLRP3 in macrophages, endothelial cells, and fibroblasts, providing more detailed theoretical support for precise targeting. With the continuous deepening of research, the NLRP3 targeted therapy strategy is expected to bring new hope to organ transplant patients, helping to achieve the goal of long-term survival of transplants and improved patient prognosis.

## References

[1] Gruessner AC Gruessner RWG . The 2022 international pancreas transplant registry report-a review. Transplant Proc. (2022) 54:1918–43. doi: 10.1016/j.transproceed.2022.03.059, PMID: 35970624

[2] Mahajan RG Evans M Kizilbash S . Kidney transplant outcomes in children with simultaneous versus sequential heart-kidney transplants. Pediatr Nephrol. (2024) 39:3095–102. doi: 10.1007/s00467-024-06412-7, PMID: 38822859

[3] Pischke S Lege MC von Wulffen M Galante A Otto B Wehmeyer MH . Factors associated with long-term survival after liver transplantation: A retrospective cohort study. World J Hepatol. (2017) 9:427–35. doi: 10.4254/wjh.v9.i8.427, PMID: 28357030 PMC5355765

[4] Odenwald MA Roth HF Reticker A Segovia M Pillai A . Evolving challenges with long-term care of liver transplant recipients. Clin Transplant. (2023) 37:e15085. doi: 10.1111/ctr.15085, PMID: 37545440

[5] Czigany Z Lurje I Schmelzle M Schöning W Öllinger R Raschzok N . Ischemia-reperfusion injury in marginal liver grafts and the role of hypothermic machine perfusion: molecular mechanisms and clinical implications. J Clin Med. (2020) 9:846. doi: 10.3390/jcm9030846, PMID: 32244972 PMC7141496

[6] Kawashima M Juvet SC . The role of innate immunity in the long-term outcome of lung transplantation. Ann Transl Med. (2020) 8:412. doi: 10.21037/atm.2020.03.20, PMID: 32355856 PMC7186608

[7] Mishra S Taneja S . Algorithmic approach to deranged liver functions after transplantation. J Clin Exp Hepatol. (2024) 14:101317. doi: 10.1016/j.jceh.2023.101317, PMID: 38264576 PMC10801315

[8] Wang Z Ge W Zhong X Tong S Zheng S Xu X . Inhibition of cysteine-serine-rich nuclear protein 1 ameliorates ischemia-reperfusion injury during liver transplantation in an MAPK-dependent manner. Mol BioMed. (2024) 5:22. doi: 10.1186/s43556-024-00185-z, PMID: 38902590 PMC11189853

[9] Poole D Skurzak S Mehra MR . Prediction of optimal outcomes in organ transplantation. Intensive Care Med. (2019) 45:367–70. doi: 10.1007/s00134-018-5472-6, PMID: 30483836

[10] Chen QD Liu L Zhao XH Liang JB Li SW . Challenges and opportunities in the islet transplantation microenvironment: A comprehensive summary of inflammatory cytokine, immune cells, and vascular endothelial cells. Front Immunol. (2023) 14:1293762. doi: 10.3389/fimmu.2023.1293762, PMID: 38111575 PMC10725940

[11] Zoller EE Lykens JE Terrell CE Aliberti J Filipovich AH Henson PM . Hemophagocytosis causes a consumptive anemia of inflammation. J Exp Med. (2011) 208:1203–14. doi: 10.1084/jem.20102538, PMID: 21624938 PMC3173248

[12] Christgen S Place DE Kanneganti TD . Toward targeting inflammasomes: insights into their regulation and activation. Cell Res. (2020) 30:315–27. doi: 10.1038/s41422-020-0295-8, PMID: 32152420 PMC7118104

[13] Jimenez-Duran G Triantafilou M . Metabolic regulators of enigmatic inflammasomes in autoimmune diseases and crosstalk with innate immune receptors. Immunology. (2021) 163:348–62. doi: 10.1111/imm.13326, PMID: 33682108 PMC8274167

[14] Du X Que W Hu X Yu X Guo WZ Zhang S . Oridonin prolongs the survival of mouse cardiac allografts by attenuating the NF-κB/NLRP3 pathway. Front Immunol. (2021) 12:719574. doi: 10.3389/fimmu.2021.719574, PMID: 34566976 PMC8462485

[15] Jin J Zhou TJ Ren GL Cai L Meng XM . Novel insights into NOD-like receptors in renal diseases. Acta Pharmacol Sin. (2022) 43:2789–806. doi: 10.1038/s41401-022-00886-7, PMID: 35365780 PMC8972670

[16] de Alba E . Structure, interactions and self-assembly of ASC-dependent inflammasomes. Arch Biochem Biophys. (2019) 670:15–31. doi: 10.1016/j.abb.2019.05.023, PMID: 31152698 PMC8455077

[17] Bracey NA Platnich JM Lau A Chung H Hyndman ME MacDonald JA . Tissue-selective alternate promoters guide NLRP6 expression. Life Sci Alliance. (2021) 4:e202000897. doi: 10.26508/lsa.202000897, PMID: 33376129 PMC7772780

[18] Hochheiser IV Behrmann H Hagelueken G Rodríguez-Alcázar JF Kopp A Latz E . Directionality of PYD filament growth determined by the transition of NLRP3 nucleation seeds to ASC elongation. Sci Adv. (2022) 8:eabn7583. doi: 10.1126/sciadv.abn7583, PMID: 35559676 PMC9106292

[19] Sandall CF MacDonald JA . Structural insights into the ATP-dependent activation of NOD-like receptor with pyrin 3 (NLRP3) protein by molecular dynamics simulation. J Biomol Struct Dyn. (2024) 43:10196–208. doi: 10.1080/07391102.2024.2434033, PMID: 39616542

[20] Atalay Ekiner S Gęgotek A Skrzydlewska E . Inflammasome activity regulation by PUFA metabolites. Front Immunol. (2024) 15:1452749. doi: 10.3389/fimmu.2024.1452749, PMID: 39290706 PMC11405227

[21] Xu B Cerbu A Tralie CJ Lim D Krasileva K . Structure-aware annotation of leucine-rich repeat domains. PloS Comput Biol. (2024) 20:e1012526. doi: 10.1371/journal.pcbi.1012526, PMID: 39499733 PMC11567593

[22] Putnam CD Broderick L Hoffman HM . The discovery of NLRP3 and its function in cryopyrin-associated periodic syndromes and innate immunity. Immunol Rev. (2024) 322:259–82. doi: 10.1111/imr.13292, PMID: 38146057 PMC10950545

[23] Sharma M de Alba E . Structure, activation and regulation of NLRP3 and AIM2 inflammasomes. Int J Mol Sci. (2021) 22:872. doi: 10.3390/ijms22020872, PMID: 33467177 PMC7830601

[24] Sharif H Wang L Wang WL Magupalli VG Andreeva L Qiao Q . Structural mechanism for NEK7-licensed activation of NLRP3 inflammasome. Nature. (2019) 570:338–43. doi: 10.1038/s41586-019-1295-z, PMID: 31189953 PMC6774351

[25] Payne FM Dabb AR Harrison JC Sammut IA . Inhibitors of NLRP3 inflammasome formation: A cardioprotective role for the gasotransmitters carbon monoxide, nitric oxide, and hydrogen sulphide in acute myocardial infarction. Int J Mol Sci. (2024) 25:9247. doi: 10.3390/ijms25179247, PMID: 39273196 PMC11395567

[26] Molla MD Ayelign B Dessie G Geto Z Admasu TD . Caspase-1 as a regulatory molecule of lipid metabolism. Lipids Health Dis. (2020) 19:34. doi: 10.1186/s12944-020-01220-y, PMID: 32143623 PMC7060649

[27] Nguyen TTM Gillet G Popgeorgiev N . Caspases in the developing central nervous system: apoptosis and beyond. Front Cell Dev Biol. (2021) 9:702404. doi: 10.3389/fcell.2021.702404, PMID: 34336853 PMC8322698

[28] Farag NS Breitinger U Breitinger HG El Azizi MA . Viroporins and inflammasomes: A key to understand virus-induced inflammation. Int J Biochem Cell Biol. (2020) 122:105738. doi: 10.1016/j.biocel.2020.105738, PMID: 32156572 PMC7102644

[29] Zheng D Liu J Piao H Zhu Z Wei R Liu K . ROS-triggered endothelial cell death mechanisms: focus on pyroptosis, parthanatos, and ferroptosis. Front Immunol. (2022) 13:1039241. doi: 10.3389/fimmu.2022.1039241, PMID: 36389728 PMC9663996

[30] Exconde PM Hernandez-Chavez C Bourne CM Richards RM Bray MB Lopez JL . The tetrapeptide sequence of IL-18 and IL-1β Regulates their recruitment and activation by inflammatory caspases. Cell Rep. (2023) 42:113581. doi: 10.1016/j.celrep.2023.113581, PMID: 38103201 PMC11158830

[31] Zheng Z Li G . Mechanisms and therapeutic regulation of pyroptosis in inflammatory diseases and cancer. Int J Mol Sci. (2020) 21:1456. doi: 10.3390/ijms21041456, PMID: 32093389 PMC7073143

[32] Wei S Ma W Zhang B Li W . NLRP3 inflammasome: A promising therapeutic target for drug-induced toxicity. Front Cell Dev Biol. (2021) 9:634607. doi: 10.3389/fcell.2021.634607, PMID: 33912556 PMC8072389

[33] McKee CM Coll RC . NLRP3 inflammasome priming: A riddle wrapped in a mystery inside an enigma. J Leukoc Biol. (2020) 108:937–52. doi: 10.1002/jlb.3mr0720-513r, PMID: 32745339

[34] Alhamdan F Bayarsaikhan G Yuki K . Toll-like receptors and integrins crosstalk. Front Immunol. (2024) 15:1403764. doi: 10.3389/fimmu.2024.1403764, PMID: 38915411 PMC11194410

[35] Duan T Du Y Xing C Wang HY Wang RF . Toll-like receptor signaling and its role in cell-mediated immunity. Front Immunol. (2022) 13:812774. doi: 10.3389/fimmu.2022.812774, PMID: 35309296 PMC8927970

[36] Wan P Zhang S Ruan Z Liu X Yang G Jia Y . AP-1 signaling pathway promotes pro-IL-1β Transcription to facilitate NLRP3 inflammasome activation upon influenza a virus infection. Virulence. (2022) 13:502–13. doi: 10.1080/21505594.2022.2040188, PMID: 35300578 PMC8942419

[37] Midtbö K Eklund D Särndahl E Persson A . Molecularly distinct NLRP3 inducers mediate diverse ratios of interleukin-1β and interleukin-18 from human monocytes. Mediators Inflammation. (2020) 2020:4651090. doi: 10.1155/2020/4651090, PMID: 33144845 PMC7599400

[38] Meng H Huan Y Zhang K Yi X Meng X Kang E . Quiescent adult neural stem cells: developmental origin and regulatory mechanisms. Neurosci Bull. (2024) 40:1353–63. doi: 10.1007/s12264-024-01206-1, PMID: 38656419 PMC11365920

[39] Stanton C Sun J Nutsch K Rosarda JD Nguyen T Li-Ma C . Covalent targeting as a common mechanism for inhibiting NLRP3 inflammasome assembly. bioRxiv. (2024) 19:254–65. doi: 10.1101/2023.06.01.543248, PMID: 38198472 PMC11131128

[40] He Y Zeng MY Yang D Motro B Núñez G . NEK7 is an essential mediator of NLRP3 activation downstream of potassium efflux. Nature. (2016) 530:354–7. doi: 10.1038/nature16959, PMID: 26814970 PMC4810788

[41] Xiao L Magupalli VG Wu H . Cryo-em structures of the active NLRP3 inflammasome disc. Nature. (2023) 613:595–600. doi: 10.1038/s41586-022-05570-8, PMID: 36442502 PMC10091861

[42] Suetomi T Willeford A Brand CS Cho Y Ross RS Miyamoto S . Inflammation and NLRP3 inflammasome activation initiated in response to pressure overload by ca(2+)/calmodulin-dependent protein kinase II δ Signaling in cardiomyocytes are essential for adverse cardiac remodeling. Circulation. (2018) 138:2530–44. doi: 10.1161/circulationaha.118.034621, PMID: 30571348 PMC6309790

[43] Wu KKL Long K Lin H Siu PMF Hoo RLC Ye D . The APPL1-rab5 axis restricts NLRP3 inflammasome activation through early endosomal-dependent mitophagy in macrophages. Nat Commun. (2021) 12:6637. doi: 10.1038/s41467-021-26987-1, PMID: 34789781 PMC8599493

[44] Li D Wang L Ou J Wang C Zhou J Lu L . Reactive oxygen species induced by uric acid promote NRK−52E cell apoptosis through the NEK7−NLRP3 signaling pathway. Mol Med Rep. (2021) 24:729. doi: 10.3892/mmr.2021.12368, PMID: 34414459 PMC8383041

[45] Akbal A Dernst A Lovotti M Mangan MSJ McManus RM Latz E . How location and cellular signaling combine to activate the NLRP3 inflammasome. Cell Mol Immunol. (2022) 19:1201–14. doi: 10.1038/s41423-022-00922-w, PMID: 36127465 PMC9622870

[46] Hornung V Bauernfeind F Halle A Samstad EO Kono H Rock KL . Silica crystals and aluminum salts activate the NALP3 inflammasome through phagosomal destabilization. Nat Immunol. (2008) 9:847–56. doi: 10.1038/ni.1631, PMID: 18604214 PMC2834784

[47] Kim MJ Yoon JH Ryu JH . Mitophagy: A balance regulator of NLRP3 inflammasome activation. BMB Rep. (2016) 49:529–35. doi: 10.5483/bmbrep.2016.49.10.115, PMID: 27439607 PMC5227293

[48] Degechisa ST Dabi YT Gizaw ST . The mitochondrial associated endoplasmic reticulum membranes: A platform for the pathogenesis of inflammation-mediated metabolic diseases. Immun Inflammation Dis. (2022) 10:e647. doi: 10.1002/iid3.647, PMID: 35759226 PMC9168553

[49] Brint A Greene S Fennig-Victor AR Wang S . Multiple sclerosis: the NLRP3 inflammasome, gasdermin D, and therapeutics. Ann Transl Med. (2024) 12:62. doi: 10.21037/atm-23-1960, PMID: 39118955 PMC11304424

[50] Ma W Wang Y Liu J . NLRP3 inflammasome activation in liver disorders: from molecular pathways to therapeutic strategies. J Inflammation Res. (2025) 18:8277–94. doi: 10.2147/jir.S532908, PMID: 40585040 PMC12205705

[51] He KL Yu X Xia L Xie YD Qi EB Wan L . A new perspective on the regulation of neuroinflammation in intracerebral hemorrhage: mechanisms of NLRP3 inflammasome activation and therapeutic strategies. Front Immunol. (2025) 16:1526786. doi: 10.3389/fimmu.2025.1526786, PMID: 40083546 PMC11903264

[52] Stutz A Kolbe CC Stahl R Horvath GL Franklin BS van Ray O . NLRP3 inflammasome assembly is regulated by phosphorylation of the pyrin domain. J Exp Med. (2017) 214:1725–36. doi: 10.1084/jem.20160933, PMID: 28465465 PMC5460996

[53] Beesetti S . Ubiquitin ligases in control: regulating NLRP3 inflammasome activation. Front Biosci (Landmark Ed). (2025) 30:25970. doi: 10.31083/fbl25970, PMID: 40152367

[54] Zhou H Feng L Xu F Sun Y Ma Y Zhang X . Berberine inhibits palmitate-induced NLRP3 inflammasome activation by triggering autophagy in macrophages: A new mechanism linking berberine to insulin resistance improvement. BioMed Pharmacother. (2017) 89:864–74. doi: 10.1016/j.biopha.2017.03.003, PMID: 28282788

[55] Wu T Zhang C Shao T Chen J Chen D . The role of NLRP3 inflammasome activation pathway of hepatic macrophages in liver ischemia-reperfusion injury. Front Immunol. (2022) 13:905423. doi: 10.3389/fimmu.2022.905423, PMID: 35757691 PMC9229592

[56] Soares ROS Losada DM Jordani MC Évora P Castro ESO . Ischemia/reperfusion injury revisited: an overview of the latest pharmacological strategies. Int J Mol Sci. (2019) 20:5034. doi: 10.3390/ijms20205034, PMID: 31614478 PMC6834141

[57] MT O Menyhárt Á Frank R Hantosi D Farkas E Bari F . Tissue acidosis associated with ischemic stroke to guide neuroprotective drug delivery. Biol (Basel). (2020) 9:460. doi: 10.3390/biology9120460, PMID: 33322264 PMC7764344

[58] Jones CE Thomas JX Parker JC Parker RE . Acute changes in high energy phosphates, nucleotide derivatives, and contractile force in ischaemic and nonischaemic canine myocardium following coronary occlusion. Cardiovasc Res. (1976) 10:275–82. doi: 10.1093/cvr/10.3.275, PMID: 954015

[59] Apichartpiyakul P Shinlapawittayatorn K Rerkasem K Chattipakorn SC Chattipakorn N . Mechanisms and interventions on acute lower limb ischemia/reperfusion injury: A review and insights from cell to clinical investigations. Ann Vasc Surg. (2022) 86:452–81. doi: 10.1016/j.avsg.2022.04.040, PMID: 35589030

[60] Dan Dunn J Alvarez LA Zhang X Soldati T . Reactive oxygen species and mitochondria: A nexus of cellular homeostasis. Redox Biol. (2015) 6:472–85. doi: 10.1016/j.redox.2015.09.005, PMID: 26432659 PMC4596921

[61] Yuan Y Shen Q Jin J . Interaction between nod-like receptor protein 3 inflammatory corpuscles and mitochondrial dysfunction in the pathogenesis of septic cardiomyopathy. Zhonghua Wei Zhong Bing Ji Jiu Yi Xue. (2024) 36:313–9. doi: 10.3760/cma.j.cn121430-20230518-00379, PMID: 38538363

[62] Boucher D Monteleone M Coll RC Chen KW Ross CM Teo JL . Caspase-1 self-cleavage is an intrinsic mechanism to terminate inflammasome activity. J Exp Med. (2018) 215:827–40. doi: 10.1084/jem.20172222, PMID: 29432122 PMC5839769

[63] Blevins HM Xu Y Biby S Zhang S . The NLRP3 inflammasome pathway: A review of mechanisms and inhibitors for the treatment of inflammatory diseases. Front Aging Neurosci. (2022) 14:879021. doi: 10.3389/fnagi.2022.879021, PMID: 35754962 PMC9226403

[64] Fu J Wu H . Structural mechanisms of NLRP3 inflammasome assembly and activation. Annu Rev Immunol. (2023) 41:301–16. doi: 10.1146/annurev-immunol-081022-021207, PMID: 36750315 PMC10159982

[65] Su Y Wang Y Liu M Chen H . Hydrogen sulfide attenuates renal I/R−Induced activation of the inflammatory response and apoptosis via regulating nrf2−Mediated NLRP3 signaling pathway inhibition. Mol Med Rep. (2021) 24:518. doi: 10.3892/mmr.2021.12157, PMID: 34013370 PMC8160482

[66] Tang TT Lv LL Pan MM Wen Y Wang B Li ZL . Hydroxychloroquine attenuates renal ischemia/reperfusion injury by inhibiting cathepsin mediated NLRP3 inflammasome activation. Cell Death Dis. (2018) 9:351. doi: 10.1038/s41419-018-0378-3, PMID: 29500339 PMC5834539

[67] Zou XF Gu JH Duan JH Hu ZD Cui ZL . The NLRP3 inhibitor mcc950 attenuates acute allograft damage in rat kidney transplants. Transpl Immunol. (2020) 61:101293. doi: 10.1016/j.trim.2020.101293, PMID: 32407873

[68] Zhang L Wang M An R Dai J Liu S Chen M . Activation of NLRP3 inflammasome via drp1 overexpression in kupffer cells aggravates ischemia-reperfusion injury in hepatic steatosis. J Clin Transl Hepatol. (2023) 11:1069–78. doi: 10.14218/jcth.2022.00109, PMID: 37577223 PMC10412692

[69] Xu L Zeng Z Niu C Liu D Lin S Liu X . Normothermic ex vivo heart perfusion with NLRP3 inflammasome inhibitor mcc950 treatment improves cardiac function of circulatory death hearts after transplantation. Front Cardiovasc Med. (2023) 10:1126391. doi: 10.3389/fcvm.2023.1126391, PMID: 37008319 PMC10063899

[70] Li G Jin B Zhou J Sun T Wang S Fan Z . Mechanisms and nanomedicine interventions of acute lung injury induced by intestinal ischemia-reperfusion: A mini review. Int J Nanomedicine. (2025) 20:9347–67. doi: 10.2147/ijn.S533797, PMID: 40735744 PMC12306567

[71] Becker S Swoboda A Siemer H Schimmelpfennig S Sargin S Shahin V . Membrane potential dynamics of C5a-stimulated neutrophil granulocytes. Pflugers Arch. (2024) 476:1007–18. doi: 10.1007/s00424-024-02947-8, PMID: 38613695 PMC11139730

[72] Herrmann JB Muenstermann M Strobel L Schubert-Unkmeir A Woodruff TM Gray-Owen SD . Complement C5a receptor 1 exacerbates the pathophysiology of N. meningitidis sepsis and is a potential target for disease treatment. mBio. (2018) 9:e01755–17. doi: 10.1128/mBio.01755-17, PMID: 29362231 PMC5784250

[73] Amores-Iniesta J Barberà-Cremades M Martínez CM Pons JA Revilla-Nuin B Martínez-Alarcón L . Extracellular ATP activates the NLRP3 inflammasome and is an early danger signal of skin allograft rejection. Cell Rep. (2017) 21:3414–26. doi: 10.1016/j.celrep.2017.11.079, PMID: 29262323 PMC5746605

[74] Que X Zheng S Song Q Pei H Zhang P . Fantastic voyage: the journey of NLRP3 inflammasome activation. Genes Dis. (2024) 11:819–29. doi: 10.1016/j.gendis.2023.01.009, PMID: 37692521 PMC10491867

[75] Potere N Garrad E Kanthi Y Di Nisio M Kaplanski G Bonaventura A . Nlrp3 inflammasome and interleukin-1 contributions to covid-19-associated coagulopathy and immunothrombosis. Cardiovasc Res. (2023) 119:2046–60. doi: 10.1093/cvr/cvad084, PMID: 37253117 PMC10893977

[76] Kanojia RP Pandey A Bawa M . Robotic assisted vesicoscopic cohen’s reimplantation in pediatric patient: nuances of technique, experience, and outcome. J Laparoendosc Adv Surg Tech A. (2020) 30:1137–41. doi: 10.1089/lap.2020.0401, PMID: 32857011

[77] He H Zhang X He H Xu G Li L Yang C . Microglial priming by IFN-Γ Involves STAT1-mediated activation of the NLRP3 inflammasome. CNS Neurosci Ther. (2024) 30:e70061. doi: 10.1111/cns.70061, PMID: 39392762 PMC11468839

[78] Wu KK Cheung SW Cheng KK . NLRP3 inflammasome activation in adipose tissues and its implications on metabolic diseases. Int J Mol Sci. (2020) 21:4184. doi: 10.3390/ijms21114184, PMID: 32545355 PMC7312293

[79] Yang D Wang Z Chen Y Guo Q Dong Y . Interactions between gut microbes and NLRP3 inflammasome in the gut-brain axis. Comput Struct Biotechnol J. (2023) 21:2215–27. doi: 10.1016/j.csbj.2023.03.017, PMID: 37035548 PMC10074411

[80] Li L Liu S Tan J Wei L Wu D Gao S . Recent advance in treatment of atherosclerosis: key targets and plaque-positioned delivery strategies. J Tissue Eng. (2022) 13:20417314221088509. doi: 10.1177/20417314221088509, PMID: 35356091 PMC8958685

[81] Vilaysane A Chun J Seamone ME Wang W Chin R Hirota S . The NLRP3 inflammasome promotes renal inflammation and contributes to CKD. J Am Soc Nephrol. (2010) 21:1732–44. doi: 10.1681/asn.2010020143, PMID: 20688930 PMC3013544

[82] Shigeoka AA Mueller JL Kambo A Mathison JC King AJ Hall WF . An inflammasome-independent role for epithelial-expressed NLRP3 in renal ischemia-reperfusion injury. J Immunol. (2010) 185:6277–85. doi: 10.4049/jimmunol.1002330, PMID: 20962258 PMC3020135

[83] You D Nie K Wu X Weng M Yang L Chen Y . C3a/C3aR synergies with TGF-β to promote epithelial-mesenchymal transition of renal tubular epithelial cells via the activation of the NLRP3 inflammasome. J Transl Med. (2023) 21:904. doi: 10.1186/s12967-023-04764-6, PMID: 38082306 PMC10714586

[84] Matsui F Rhee A Hile KL Zhang H Meldrum KK . IL-18 induces profibrotic renal tubular cell injury via STAT3 activation. Am J Physiol Renal Physiol. (2013) 305:F1014–21. doi: 10.1152/ajprenal.00620.2012, PMID: 23904224 PMC4073972

[85] Zohorsky K Lin S Mequanint K . Immobilization of jagged1 enhances vascular smooth muscle cells maturation by activating the notch pathway. Cells. (2021) 10:2089. doi: 10.3390/cells10082089, PMID: 34440858 PMC8391929

[86] Burger F Baptista D Roth A da Silva RF Montecucco F Mach F . NLRP3 inflammasome activation controls vascular smooth muscle cells phenotypic switch in atherosclerosis. Int J Mol Sci. (2021) 23:340. doi: 10.3390/ijms23010340, PMID: 35008765 PMC8745068

[87] Burke RM Dale BL Dholakia S . The NLRP3 inflammasome: relevance in solid organ transplantation. Int J Mol Sci. (2021) 22:10721. doi: 10.3390/ijms221910721, PMID: 34639062 PMC8509131

[88] Li Y Qiang R Cao Z Wu Q Wang J Lyu W . NLRP3 inflammasomes: dual function in infectious diseases. J Immunol. (2024) 213:407–17. doi: 10.4049/jimmunol.2300745, PMID: 39102612 PMC11299487

[89] Huang X Sun P Qin Y Wang XJ Wang M Lin Y . Disulfiram attenuates MCMV-induced pneumonia by inhibition of NF-κB/NLRP3 signaling pathway in immunocompromised mice. Int Immunopharmacol. (2022) 103:108453. doi: 10.1016/j.intimp.2021.108453, PMID: 34959186

[90] Sen P Wilkie AR Ji F Yang Y Taylor IJ Velazquez-Palafox M . Linking indirect effects of cytomegalovirus in transplantation to modulation of monocyte innate immune function. Sci Adv. (2020) 6:eaax9856. doi: 10.1126/sciadv.aax9856, PMID: 32494628 PMC7176434

[91] Moretti J Blander JM . Increasing complexity of NLRP3 inflammasome regulation. J Leukoc Biol. (2021) 109:561–71. doi: 10.1002/jlb.3mr0520-104rr, PMID: 32531835 PMC8985609

[92] Jiang C Xie S Yang G Wang N . Spotlight on NLRP3 inflammasome: role in pathogenesis and therapies of atherosclerosis. J Inflammation Res. (2021) 14:7143–72. doi: 10.2147/jir.S344730, PMID: 34992411 PMC8711145

[93] Xu W Huang Y Zhou R . NLRP3 inflammasome in neuroinflammation and central nervous system diseases. Cell Mol Immunol. (2025) 22:341–55. doi: 10.1038/s41423-025-01275-w, PMID: 40075143 PMC11955557

[94] Peng L Jiang J Chen T Xu D Hou F Huang Q . Toxic shock syndrome toxin 1 induces immune response via the activation of NLRP3 inflammasome. Toxins (Basel). (2021) 13:68. doi: 10.3390/toxins13010068, PMID: 33477467 PMC7829800

[95] Hou F Peng L Jiang J Chen T Xu D Huang Q . ATP facilitates staphylococcal enterotoxin O induced neutrophil IL-1β Secretion via NLRP3 inflammasome dependent pathways. Front Immunol. (2021) 12:649235. doi: 10.3389/fimmu.2021.649235, PMID: 34017331 PMC8129502

[96] Lee HM Yuk JM Kim KH Jang J Kang G Park JB . Mycobacterium abscessus activates the NLRP3 inflammasome via dectin-1-syk and P62/SQSTM1. Immunol Cell Biol. (2012) 90:601–10. doi: 10.1038/icb.2011.72, PMID: 21876553 PMC3389799

[97] Hu K Jiang X Zhang J Xia D Wu D Shao J . Effect of pulsatilla decoction on vulvovaginal candidiasis in mice. Evidences for its mechanisms of action. Phytomedicine. (2024) 128:155515. doi: 10.1016/j.phymed.2024.155515, PMID: 38484624

[98] Thrikawala SU Anderson MH Rosowski EE . Glucocorticoids suppress NF-κB-mediated neutrophil control of aspergillus fumigatus hyphal growth. J Immunol. (2024) 213:971–87. doi: 10.4049/jimmunol.2400021, PMID: 39178124 PMC11408098

[99] Singh N Huprikar S Burdette SD Morris MI Blair JE Wheat LJ . Donor-derived fungal infections in organ transplant recipients: guidelines of the american society of transplantation, infectious diseases community of practice. Am J Transplant. (2012) 12:2414–28. doi: 10.1111/j.1600-6143.2012.04100.x, PMID: 22694672

[100] Gaziano R Sabbatini S Monari C . The interplay between candida albicans, vaginal mucosa, host immunity and resident microbiota in health and disease: an overview and future perspectives. Microorganisms. (2023) 11:1211. doi: 10.3390/microorganisms11051211, PMID: 37317186 PMC10222832

[101] Banerjee SK Chatterjee A Gupta S Nagar A . Activation and regulation of NLRP3 by sterile and infectious insults. Front Immunol. (2022) 13:896353. doi: 10.3389/fimmu.2022.896353, PMID: 35663964 PMC9161712

[102] Jiang Q Chen Y Zheng S Sui L Yu D Qing F . AIM2 enhances candida albicans infection through promoting macrophage apoptosis via AKT signaling. Cell Mol Life Sci. (2024) 81:280. doi: 10.1007/s00018-024-05326-9, PMID: 38918243 PMC11335202

[103] Yuan B Shen H Lin L Su T Zhong S Yang Z . Recombinant adenovirus encoding NLRP3 RNAi attenuate inflammation and brain injury after intracerebral hemorrhage. J Neuroimmunol. (2015) 287:71–5. doi: 10.1016/j.jneuroim.2015.08.002, PMID: 26439964

[104] Coll RC Robertson AA Chae JJ Higgins SC Muñoz-Planillo R Inserra MC . A small-molecule inhibitor of the NLRP3 inflammasome for the treatment of inflammatory diseases. Nat Med. (2015) 21:248–55. doi: 10.1038/nm.3806, PMID: 25686105 PMC4392179

[105] Zhang C Zhu X Li L Ma T Shi M Yang Y . A small molecule inhibitor mcc950 ameliorates kidney injury in diabetic nephropathy by inhibiting NLRP3 inflammasome activation. Diabetes Metab Syndr Obes. (2019) 12:1297–309. doi: 10.2147/dmso.S199802, PMID: 31447572 PMC6684489

[106] Cao N Wang JJ Wu JM Xu WL Wang R Chen XD . Glibenclamide alleviates β Adrenergic receptor activation-induced cardiac inflammation. Acta Pharmacol Sin. (2022) 43:1243–50. doi: 10.1038/s41401-021-00734-0, PMID: 34349235 PMC9061800

[107] Chen LL Song C Zhang Y Li Y Zhao YH Lin FY . Quercetin protects against LPS-induced lung injury in mice via SIRT1-mediated suppression of PKM2 nuclear accumulation. Eur J Pharmacol. (2022) 936:175352. doi: 10.1016/j.ejphar.2022.175352, PMID: 36309049

[108] Yin H Guo Q Li X Tang T Li C Wang H . Curcumin suppresses IL-1β Secretion and prevents inflammation through inhibition of the NLRP3 inflammasome. J Immunol. (2018) 200:2835–46. doi: 10.4049/jimmunol.1701495, PMID: 29549176

[109] Yin Y Tang L Liu K Ding X Wang D Chen L . Attenuation of lipopolysaccharide-induced liver injury by bone marrow mesenchymal stem cells via inhibiting the nlrp3 inflammasome and hepatocyte pyroptosis. Curr Stem Cell Res Ther. (2022) 17:361–9. doi: 10.2174/1574888x17666220407103441, PMID: 35392791

[110] Yao S Pang M Wang Y Wang X Lin Y Lv Y . Mesenchymal stem cell attenuates spinal cord injury by inhibiting mitochondrial quality control-associated neuronal ferroptosis. Redox Biol. (2023) 67:102871. doi: 10.1016/j.redox.2023.102871, PMID: 37699320 PMC10506061

[111] Li H Wang C He T Zhao T Chen YY Shen YL . Mitochondrial transfer from bone marrow mesenchymal stem cells to motor neurons in spinal cord injury rats via gap junction. Theranostics. (2019) 9:2017–35. doi: 10.7150/thno.29400, PMID: 31037154 PMC6485285

[112] Deaglio S Dwyer KM Gao W Friedman D Usheva A Erat A . Adenosine generation catalyzed by CD39 and CD73 expressed on regulatory T cells mediates immune suppression. J Exp Med. (2007) 204:1257–65. doi: 10.1084/jem.20062512, PMID: 17502665 PMC2118603

[113] Dai H Pena A Bauer L Williams A Watkins SC Camirand G . Treg suppression of immunity within inflamed allogeneic grafts. JCI Insight. (2022) 7:e160579. doi: 10.1172/jci.insight.160579, PMID: 35881490 PMC9462475

[114] Gauthier JM Li W Kreisel D . Commentary: T cells regulate lung transplant rejection in mice and men. J Thorac Cardiovasc Surg. (2019) 157:2538–9. doi: 10.1016/j.jtcvs.2019.01.101, PMID: 30824351

